# Role of gamma nail in management of pertrochanteric fractures of femur

**DOI:** 10.4103/0019-5413.40260

**Published:** 2008

**Authors:** Vipin Sharma, Sushrut Babhulkar, Sudhir Babhulkar

**Affiliations:** Dr. Rajendra Prasad Medical College and Hospital, Kangra at Tanda (HP), India; 1 Sushrut Hospital, Research Centre and Post Graduate Institute of Orthopedics/Indira Gandhi Medical College, Nagpur, India

**Keywords:** Gamma nail, pertrochanteric fractures

## Abstract

**Background:**

Pertrochanteric fractures which involve trochanteric fractures with varying fracture geometry pose a significant challenge to the treating orthopedic surgeon. The aim of this study is to evaluate the management of pertrochanteric fractures of the femur using gamma nail [Asia pacific (AP)].

**Materials and Methods:**

Sixty patients of pertrochanteric fractures were treated by closed reduction internal fixation by gamma nail from 1 January 1993 to 31 December 2000. Four patients were lost to follow-up. The remaining 56 patients were followed for a mean period of 3.2 years (range 2-4 years).The results were evaluated by assessing the patients regarding their clinical and functional outcome at follow-up as per Kyle's criteria.

**Results:**

Peroperative jamming of nail (*n* = 1), failed distal locking (*n* = 1), superior cut out of lag screw (*n* = 1) and postoperative varus malreduction (*n* = 1) were the complications observed. End results were excellent in 46.34%, good in 36.58%, fair in 14.64%, poor in 2.43%.

**Conclusion:**

Gamma nail in expert hands is a suitable implant for management of pertrochanteric fractures of the femur.

## INTRODUCTION

Gamma nail is a versatile implant for fixation of pertrochanteric fractures which include trochanteric fractures of different geometry. Development of this nail progressed through various designs. Initial design was called as Mark I. Subsequent designs that followed were called Mark II and Mark III. Initially it was called Halifax Nail after the place where it was developed by Dr. Subhash Haldar.^1^ A group of surgeons from Strasbourg^2^ changed the name of this nail to a universal one i.e. Gamma Nail as the shape resembled the Greek letter.^1^

But these initial designs were associated with a host of peroperative complications when applied to Asiatic femora like jamming of nail, impingment of tip of nail on the anterior cortex and fracture of lateral cortex of femur. K. S. Leung in Hong Kong undertook an anthropometeric study on Asiatic femora to circumvent these complications and brought out a design called Asia Pacific gamma nail.^3^

The comparative dimensions of the standard Gamma nail and the Asia Pacific (AP) gamma nail are as shown in Table 1.^4^

**Table 1:** Comparative Dimensions of Gamma Nail

Geometric parameters	Standard	Modified (Asia Pacific)
Length (mm)	200	180
distal diameter (mm)*	12,14,16	11,12
Nail valgus (degrees)	10	4
Position of lag screw from nail tip (mm)	45	55

* millimeter

With this modified nail design nearly 60% of fractures could be fixed with lesser degree of reaming. Decreased mediolateral nail valgus together with shortened nail length decreased stress on the anterior, medial and lateral cortices. This led to decreased hoop stresses inside the canal leading to less per and postoperative complications.

Though pertrochanteric fractures have been treated in the past by a variety of fixation devices, the present study was carried out by managing these fractures by gamma nail (Asia Pacific).

## MATERIALS AND METHODS

Sixty patients suffering from pertrochanteric fractures (A1,A2 and A3 Fractures, AO Classification) were treated by gamma nail (Asia Pacific) from 1 January 1993 to 31 December 2000. The pertrochanteric fractures taken for study were fresh fractures which were studied prospectively after taking due consent. Subsequently they were subjected to management by gamma nail surgery in a consecutive fashion over the above mentioned period. The study excluded patients with combination of trochanteric fractures and ipsilateral shaft fracture which were treated by long gamma nail and also those who were lost to follow-up. Patients were seen postoperatively at regular intervals of first month, third month, sixth month and then annually. All the patients were evaluated for peroperative parameters like duration of screening time (in seconds), operating time (in minutes), blood loss during surgery (in mililitres), ease of procedure; possible intraoperative complications like malreduction/failure of reduction, jamming of nail, drill breakage, failed distal locking, iatrogenic fracture shaft femur, fracture displacement. Blood loss during surgery included blood loss due to fracture and operative losses. Here screening time meant the time during which a particular fracture was screened under image intensifier during surgery. In the present study the ease of operation was categorized as easy, usual and difficult. This was purely a peroperative subjective criterion and the opinion of the operating surgeons was taken into account to label a surgery as easy, usual or difficult. Postoperatively they were assessed for malunion, delayed union, osteonecrosis of head of femur, osteoarthritic changes at hip, general and local complications and any additional /revision surgery required. Also, they were assessed for date out of bed to chair, state of ambulation, ambulatory status at discharge, requirement of ambulatory assistant devices, weight-bearing status at discharge and length of hospital stay. Radiographic assessment of fracture fragment position, lag screw position, nail alignment and extent of fracture healing was made. Overall outcome was assessed, categorizing the result as Excellent, Good, Fair and Poor as [per Kyle's criteria^5^] [Table 2].

**Table 2:** Clinical outcome (Kyle *et al.*)^5^

Excellent
No or minimal limp
No pain hip joint
Full ROM hip joint
Good
Mild limp
Mild occasional pain
Full ROM
Fair
Limp up to moderate
Moderate pain (using two sticks)
Limited ROM
Poor
Wheelchair-bound
Pain on any position
Non-ambulatory

ROM-Range of motion

### Operative Procedure

After preoperative assessment patient was taken on the traction table under spinal anesthesia. On an average one senior surgeon and two assistant surgeons participated in each surgery. Maintaining traction, closed reduction was achieved by applying slight traction in anatomic axis of the limb without any abduction or adduction and slight internal rotation or external rotation depending on underlying fracture geometry. The trunk was tilted to the unaffected side to allow access to the trochanteric area. The opposite limb was kept in flexion and abduction so as to position C-Arm. Reduction was verified on image intensifier TV control. The tip of the greater trochanter was identified by palpation and a 5-cm incision extended proximally from it. Care was taken not to extend the incision too proximally as this would damage the inferior gluteal nerve. Incision was deepened through fascia lata, splitting the abductor muscle for approximately 3 cm immediately above the tip of the greater trochanter, thus exposing its tip. Leung *et al.*,^4^ have modified the concept of reaming of gamma nail (AP). They have recommended minimal intramedullary reaming. The entry site was opened up with a cannulated curved awl and a guide wire passed into the medulla simultaneously achieving reduction at fracture site. An anteversion guide wire was placed to judge the plane of femoral neck anteversion. Reaming was done in 0.5 mm increments up to 10-12 mm with the help of flexible reamers. In order to accommodate the proximal end of the nail, the trochanteric region was reamed up to 17 mm irrespective of distal diameter chosen. Short gamma nail AP was used in all cases. Nail of chosen size was mounted on introducer jig. Nail was then passed manually with rocking motion. The incision was made on the skin overlying the lateral cortex in line with slot in proximal jig for introduction of lag screw. Lateral cortex was pierced by an awl. A guide wire was passed through guide sleeve across the lateral cortex into the posteroinferior sector of femoral head under image intensifier control and an appropriately sized lag screw was inserted after drilling over lag screw guide wire and was introduced deep in the subchondral region in the centre of head in antero posterior and lateral plane. Distal locking was done through jig or by free-hand technique under image intensifier TV control. Distally both the screws were locked to achieve rotational stability.

Immediately postoperatively patient was closely observed for vital parameters, soakage of dressing and efficiency of suction drain. Patient was kept on antibiotics for 10-12 days. Wound inspection was done in case there was postoperative fever, wound discharge and other signs of infection.

On the first postoperative day patients were made to sit up in bed and chest physiotherapy was started. Active knee bending and static quadriceps drill was also started. Toe touch weight-bearing with crutches was encouraged.

All A1 and A2 fracture at the end of first week and A3 fractures at the end of 2^nd^ week were allowed full weight-bearing with crutches followed by squatting (6-8 weeks) cross-leg setting (8-12 weeks) and full normal activity that was permitted by 15-16 weeks.

**Figure 1:**
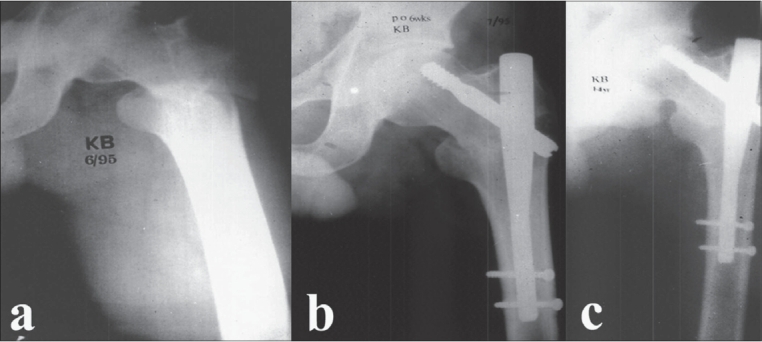
X-ray left hip antero-posterior view (a) Trochanteric fractures (A3/AO classification) left femur showing reverse obliquity. (b) Postoperative radiograph showing fracture well fixed with gamma nail showing good alignment and position. (c) 2 year follow up radiograph showing consolidation at fracture site

**Figure 2:**
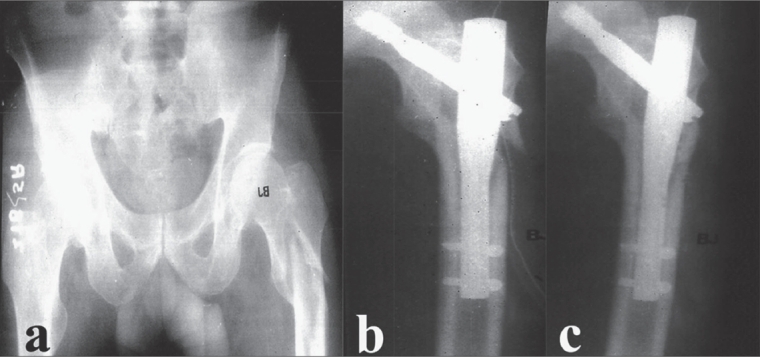
Radiograph showing comminuted trochanteric fracture (A3/AO classification) left femur. (b) Immediate postoperative radiograph showing good fracture alignment and reduction with gamma nail. (c) 1 year follow up showing good union at fracture site

Out of a total of 60 patients four were lost to follow-up before one year after injury and were excluded from the study. The remaining 56 patients were followed up for a mean period of 3.2 years (range 2-4 years). There were 52men and four women. Trochanteric fractures were classified according to AO classification into A1, A2 and A3 fractures [(A1,A2 (*n* = 36), A3 (*n* = 20)]. The mean age for AI, A2 fractures (*n* = 36) was 53 ± 5.66 years, while the mean age for A3 fractures (*n* = 20) was 29.7 ± 7.03 years. Out of a total of 56 patients, 29 suffered from high-energy trauma while 27 suffered from low-energy trauma. It was observed from the study that high-energy trauma was significant statistically (X^2^ = 18.19, *p* < 0.001) in causation of A3 fractures as compared to A1, A2 fractures. All 56 patients of pertrochanteric fractures underwent operative intervention which resulted in satisfactory outcome. The mean duration of hospital stay was 14 ± 0.72 days. Mean screening time was 31s (range 28-39 s) [Figures 1–2].

Out of a total of 56 pertrochanteric fractures three were found to be in difficult category [A2 (*n* = 2), A3 (*n* = 1)] while the rest were easy and usual. The mean operating time for these fractures was 40 min (range 35-42 min). The average blood loss during the surgery was 800 ml in A1 fractures and 850 ml in A2 and A3 fractures. Regarding postoperative mobilization all patients were shifted from bed to chair after one day. In all fractures toe touch weight-bearing was started the first postoperative day onwards. Full weight-bearing was started by the end of the first week in A1, A2 patients (*n* = 36, 64.5%). In A3 patients (*n* = 20, 35.6%) full weight-bearing was delayed till the end of the second week. This decision was taken considering the stability of nail bone construct and as per the discretion of operating surgeons. The stability of nail bone construct was assessed depending on comminution of posteromedial cortex, reversal of obliquity of the underlying fractures, the subsequent ability to achieve anatomic or near anatomic reduction and adequacy of fixation. During peroperative period there was jamming of nail (*n* = 1, 1.78%) and failed distal locking (*n* = 1, 1.78%). Jamming of nail was corrected by extracting the nail and passing a nail with lesser diameter. There was no complication like drill breakage, iatrogenic fracture shaft femur or displacement of fracture. Postoperatively one patient (1.78%) suffered from chest complications, one (1.78%) from urinary tract infection and two (3.57%) from deep venous thrombosis. One patient (1.78%) suffered from early wound infection. There was one incidence (1.78%) of superior cutout of lag screw. This was due to technical failure secondary to nonradiolucent proximal jig which led to slightly posterior placement of hip screw with a few proximal threads cutting out of articular surface. This was corrected by revision surgery five days later. The patient was taken to the operation theatre and screw was taken out. Then lag screw of appropriate size was inserted. There was varus deformity in one patient (1.78%) secondary to inappropriate placement of hip screw. This was not corrected as it was accepted by the patient. There was no incidence of implant breakage or symptomatic removal of hardware. A1 fractures achieved union by a mean period of six weeks. Fractures in A2 and A3 category took a mean period of eight weeks to unite.

The end results were found to be Excellent in 46.34%, Good in 36.58%, Fair in 14.64% and Poor in 2.43% of patients [Table 3]. There was no statistically significant difference (X^2^ = 5.61 p > 0.05) between A1, A2 and A3 fractures in terms of end result. At a mean follow-up of 3.2 years, the fractures had healed in all the patients and no further treatment was required.

**Table 3:** End result

	Excellent	Good	Fair	Poor
A1A2 fractures	14	17	5	0
A3 fractures	12	4	3	1

## DISCUSSION

Pertrochanteric fractures are one of the most commonly suffered fractures by patients of different age groups. The available published literature in this regard has shown these fractures being treated by a variety of devices like Nail Plate devices, Dynamic hip screw (DHS) and Medullary devices, e.g. Ender's Nail, Zickel nail, Gamma nail devices. Arnout *et al.*,^6^ presented 76 cases of trochanteric fractures treated by gamma nail and found it to be better than contemporary devices like Ender's nails. They found it to be especially useful in subtrochanteric fractures. Calvert^7^ in his studies found gamma nail to be better for the management of complex pertrochanteric fractures with subtrochanteric extension. Various other studies^8^–^11^ found favorable results with gamma nail in managing a greater variety of hip fractures with a less invasive technique and with better results. In the available literature the mean age for these fractures was 80 years.^3^ The preoperative screening time as reported by Leung^3^ for his pertrochanteric fractures was identical to the screening time in our study and was lesser than his screening time for pertrochanteric fractures treated by DHS. Operating time in our study was a mean period of 40 min in all fractures (range 35-42 min). This was almost identical to operation time for fractures treated by gamma nail by Leung^3^ and was much less than his operation time for DHS. The blood loss suffered by patients in our study was comparable to other series.^3^,^10^ Peroperative complications like jamming of nail, drill breakage, failed distal locking, superior cutout of lag screw and iatrogenic fracture femur were found to be much less in our group than that reported in other series.^3^,^12^,^13^ This was because the nail used by us was AP gamma nail, which has lower complication rate as compared to standard gamma nail as it conforms better to Asiatic femora. In the present study varus malunion was found to occur in one patient. The results were better than results by Leung^3^ as compared to his gamma nail and DHS group. The results regarding postoperative weight-bearing were comparable to other series.^3^,^14^ There was no mortality six months after fixation with gamma nail in the present study while Leung reported significant mortality in both his gamma nail and DHS group. There was incidence of early wound infection in one patient which was much less than in the study by Leung.^3^ The study^3^,^15^ showed a higher incidence of superior cutout of lag screw than the present study. There was only one patient (1.78%) of superior cutout of lag screw in the present study. For superior cutout of lag screw Leung reported two patients (2.2%) in the gamma nail group and three patients (3.2%) in the DHS group.^3^ The study^15^ showed this incidence to be 2% in the gamma nail group and 3% in the DHS group. Other studies^3^,^14^,^15^ showed higher incidence of fracture shaft femur in the gamma nail group but no fracture shaft femur was found in our group probably because of using gamma nail AP in our study. In the present study only one patient (1.78%) with superior cutout of lag screw underwent revision surgery while in another study^3^ revision surgery incidence in the DHS group was 7.7% and in the gamma nail group was 5.7%. In other studies^14^–^16^ revision rates after gamma nail surgery were found to be 8.8%, 6% and 8.3% respectively. These rates were very high as compared to our study. The lesser incidence of revision surgery in the present study could be due to the use of modified gamma nail (AP) and a good experience of our surgeons. Other recent studies have reported favorable results with gamma nail in terms of short operation time, less blood loss, short hospital stay, decreased wound infection and reduced complication rate.^16^–^18^ Also, it has been observed that the rate of complications associated with gamma nail decreases appreciably with increase in learning curve of the operating surgeons.^19^

## CONCLUSION

Asia Pacific gamma nail is a versatile implant for fixation of pertrochanteric fractures of the femur in the hands of surgeons who are well familiar with its technique. It gives advantage of closed technique, allows early mobilization and early weight-bearing. It involves lesser perioperative complications. Rehabilitation is easier with this device. Proximal lag screw insertion allows dynamic osteosynthesis at fracture site.
